# Resistance to gemcitabine is mediated by the circ_0036627/miR‐145/S100A16 axis in pancreatic cancer

**DOI:** 10.1111/jcmm.18444

**Published:** 2024-06-24

**Authors:** Shuo Yu, Min Wang, Hang Zhang, Xingjun Guo, Renyi Qin

**Affiliations:** ^1^ Department of Biliary‐Pancreatic Surgery Affiliated Tongji Hospital, Tongji Medical College, Huazhong University of Science and Technology Wuhan Hubei China

**Keywords:** circ_0036627/miR‐145/S100A16, gemcitabine, pancreatic cancer

## Abstract

The development of gemcitabine (GEM) resistance severely limits the treatment efficacy in pancreatic cancer (PC) and increasing evidence highlights the vital roles of circular RNAs (circRNAs) in the tumorigenesis, progression and drug resistance of PC. However, the circRNAs underlying GEM resistance development of PC remains to be clarified. The current research aims to unveil the roles of circ_0036627 in dictating the aggressiveness and GEM sensitivity in PC. We reported the increased expression of circ_0036627 in PC tissues and PC cell lines. Elevated circ_0036627 expression level was correlated with advanced tumour grade and poor overall survival in PC patients. Functional assays and *in vivo* experiments demonstrated that circ_0036627 overexpression was required for the proliferation, migration invasion and GEM resistance in PC cells. circ_0036627 knockdown suppressed tumour development in vivo. The molecular analysis further showed that circ_0036627 increased S100A16 expression by sponging microRNA‐145 (miR‐145), a tumour‐suppressive miRNA that could significantly attenuate PC cell proliferation, migration, invasion and GEM resistance. Furthermore, our findings suggested that S100A16 acted as an oncogenic factor to promote aggressiveness and GEM resistance in PC cells. In conclusion, the current findings provide new mechanistic insights into PC aggressiveness and GEM resistance, suggesting the critical role of circ_0036627/miR‐145/S100A16 axis in PC progression and drug resistance development and offering novel therapeutic targets for PC therapy.

## 1 INTRODUCTION

Pancreatic cancer (PC) is one of the most prevalent malignancies and leading causes of tumour‐related deaths globally.^1^ Although there are a variety of therapeutic options for the clinical management of PC, including surgery, chemotherapy and radiation therapy, PC remains as one of the most aggressive malignancies with an unfavourable prognosis.^1^ PC patients are often subjected to chemotherapy, with gemcitabine (GEM) serving as the standard treatment agent.^2^ The combination of GEM with other anti‐cancer drugs (including paclitaxel) has been recently established to improve patient survival.^3^ Unfortunately, a large fraction of PC patients respond poorly to existing therapies and this is owing to the fact that the PC cells undergo rapid progression and develop cellular resistance to chemotherapeutic agents.^4^ Identifying chemoresistance‐associated molecules and pathways is crucial for the formulation of novel strategies to improve treatment outcomes.

The complicated aetiology of PC tumorigenesis and chemoresistance has been revealed through the application of next‐generation sequencing technology.^5^, ^6^ Previous studies have demonstrated that mutations in different genes such as K‐RAS, hENT1, DCK, CDA, HMLH1, TP1DPD, HER2 and SMAD4, could drive the development of PC chemoresistance.^7^ However, the identified genetic alterations cannot fully explain the chemoresistance observed in PC and mounting data suggested that non‐genetic mechanisms, including microRNAs (miRNAs) and circular RNAs (circRNAs), are also implicated in chemoresistance development and treatment response.^8^ For instance, increasing number of circRNAs have been involved in PC chemoresistance through high‐throughput sequencing studies.^9^ By comparing the expression profiles of circRNAs between GEM‐resistant and parental cells, multiple circRNAs showed differential expression between GEM‐resistant PC cells and GEM‐responsive parental cells.^9^, ^10^ For example, the silencing of two novel circRNAs restored the sensitivity of PC cells to GEM treatment and the overexpression augmented the resistance to GEM,^9^ indicating that circRNAs are potential therapeutic targets for manipulating GEM resistance.

A prior study showed that circ_0036627 expression was higher in highly invasive PC cells and silencing circ_0036627 impaired the invasive potential in PC cells.^11^ Further analysis revealed that circ_0036627 functions as a sponge for miR‐338 to impinge on the invasive phenotype of PC cells.^11^ However, the potential role of circ_0036627 in dictating PC chemoresistance remains unknown. In the present report, we reported the upregulation of circ_0036627 in PC tissue and cancerous cell lines, and its overexpression was correlated with a poorer prognosis in PC patients. Overexpression of circ_0036627 increased cell growth, invasion, as well as GEM resistance by targeting the miR‐145/S100A16 axis. These results provide molecular insights into GEM resistance development and offer therapeutic targets for PC management.

## 2 MATERIALS AND METHODS

### 2.1 Cell lines

Human PC cells (AsPC‐1, PANC‐1 and PaCa‐2) and immortalized pancreatic duct epithelial cell line HPDE6‐C7 were obtained from the China Academia Sinica Cell Repository (Shanghai, China). All the cells were cultured in Dulbecco's modified Eagle's medium (DMEM, Thermo Fisher Scientific, Waltham, MA, USA) with 10% fetal bovine serum (FBS, Thermo Fisher Scientific) at 37°C and 5% CO_2_ in a humidified incubator.

### 2.2 Clinical samples

The Ethics Committee of Affiliated Tongji Hospital, Tongji Medical College, Huazhong University of Science and Technology approved the usage of human samples in this study. Sixty pairs of PC specimens and adjacent non‐tumour tissues were harvested from PC patients undergoing surgical resection at Affiliated Tongji Hospital. Informed consent was acquired from each patient. Before the operation, the patients had not received preoperative chemotherapy or radiotherapy. The tissue samples were immediately stored at −80°C until further analysis. The tissue samples were verified by two independent pathologists through histological analysis.

### 2.3 RNA extraction and real‐time PCR

Total RNA was extracted using RNAiso Plus (TaKaRa, Tokyo, Japan) according to the manufacturer's instructions. To detect circ_0036627 levels, the RNase R (Epicentre Technologies, Madison, WI, USA) was used to degrade linear RNAs before amplification. PrimeScript RT Master Mix was then employed to produce cDNA (TaKaRa). Real‐time PCR amplification was carried out using the SYBR green master mix (TaKaRa), with GAPDH as the endogenous control. For miR‐145 detection, genomic DNA was first removed and then cDNA was synthesized using Mir‐X miRNA first‐strand synthesis kit (Takara), with snRNA U6 being used to normalize the expression level of miR‐145. To detect the relative abundance of genes in the nucleus and cytoplasm, the nuclear and cytosolic fractions of PC cells were collected using the PARIS Kit (Thermo Fisher Scientific, Waltham, MA, USA) before RNA extraction. The primer sequences were shown as below: hsa_circ_0036627, forward (F): 5'‐GATGTGTCCAATCCCTGCCG‐3' and hsa_circ_0036627, reverse (R): 5'‐GCGCCTCAATTGTCATGGAA‐3; GAPDH, F: 5'‐AATCCCATCACCATCTTC‐3' and GAPDH, R: 5'‐AGGCTGTTGTCATACTTC‐3; PDE8A, F: 5'‐AAAACCCCAACATCATGGCCT‐3' and PDE8A, R: 5'‐CCTGAGTTTCAGTTGTGATCGC‐3; S100A16, F: 5'‐ATGTCAGACTGCTACACGGAG‐3' and S100A16, R: 5'‐GTTCTTGACCAGGCTGTACTTAG‐3; miR‐145, F: 5'‐CGGTCCAGTTTTCCCAGGA‐3' and miR‐145, R: 5'‐AGTGCAGGGTCCGAGGTATT‐3'; U6, F: 5'‐GCTTCGGCAGCACATATACTAAAAT‐3' and U6, R: 5'‐CGCTTCACGAATTTGCGTGTCAT‐3'.

### 2.4 Actinomycin D treatment

Actinomycin D treatment was applied to detect the stability of circ_0036627. In brief, 2 μg/mL of Actinomycin D (Sigma‐Aldrich, St. Louis, MO, USA) was used to treat 10 μg RNA at 37°C for 30 min, with the DMSO treatment as the control. The relative abundance of circ_0036627 was examined by qRT‐PCR.

### 2.5 Vectors, oligonucleotides and transfection

The circ_0036627 expression vector, the S100A16 expression vector, the negative control vector, the oligonucleotides including miR‐145 mimics, control mimics, miR‐145 inhibitor, control inhibitor, two siRNAs targeting S100A16 (si‐S100A16), and negative control siRNA (si‐NC) were purchased from RiboBio (Guangzhou, China). Lipofectamine 3000 reagent (Thermo Fisher Scientific) was used to transfect cells for 48 h before experiments. Briefly, cells were seeded in 6‐well plates at a density of 5 × 10^5^ cells/well. 100 nm of each molecule or 4 μg plasmid was mixed with 100 μL Opti‐MEM® I Reduced‐Serum Medium (Invitrogen, Carlsbad, CA, USA), and then 6 μL Lipofectamine 3000 reagent was added for 10 min incubation at room temperature. The mixture was added dropwise into the cell culture and the transfection was performed for 48 h before further experiments.

To generate cells with stable circ_0036627 knockdown, the lentivirus containing short hairpin RNA (shRNA) against circ_0036627 or negative control shRNA (sh‐NC) was obtained from GeneChem (Shanghai, China). Cells were infected with lentiviral particles for 48 h and puromycin (1 μg/mL, Sigma‐Aldrich) was used to select stably infected cells.

### 2.6 CCK‐8 cell proliferation and viability assay

Cell Counting Kit‐8 (CCK‐8, Dojindo, Japan) was used to assess cell viability. Cells were seeded into 96‐well plates at a density of 2000 cells per well, with triplicate in each experimental condition. For proliferation assay, cells were cultivated for 1–4 days. GEM was introduced in each well at increasing concentrations for 24 h to detect cellular response to GEM. At the indicated time point, 10 μL of CCK‐8 solution was added to each well for 2 h incubation. The absorbance at 450 nm was measured with a microplate reader (BioTek, Winooski, VT, USA).

### 2.7 Wound‐healing assay

In 6‐well plates, cells (1 × 10^5^) were seeded and grown to reach 95% confluence. A 200 μL plastic pipette tip was utilized to generate the wounds in the central region of the cell monolayer. Cells were grown in DMEM containing Mitomycin C (5 μg/mL, Sigma‐Aldrich) after being washed with PBS. Wounded areas were imaged using phase‐contrast microscopy immediately and at 48 h after wound creation.

### 2.8 Transwell invasion assay

Matrigel invasion assay was performed using 24‐well transwell cell culture inserts (8 μm pores, Corning, Corning, NY, USA). Cells (5 × 10^4^) suspended in 500 μL of serum‐free DMEM were seeded into the upper chamber of a 24‐well plate coated with Matrigel Matrix (Corning). The lower chamber was filled with 750 μL of DMEM with 10% FBS. The cells were cultured at 37°C for 24 h, and cells were fixed for 30 min with 4% paraformaldehyde (Sigma‐Aldrich) and stained with 0.1% crystal violet (Sigma‐Aldrich) for 20 min. The number of invading cells was counted using an inverted microscope, and 10 randomly selected fields were counted in each sample.

### 2.9 Xenograft experiment

All animal studies were conducted with the approval of the Animal Use and Care Committee of Affiliated Tongji Hospital, Tongji Medical College, Huazhong University of Science and Technology. Four‐week‐old BALB/c nude mice were provided by the SLAC Laboratory Animal Centre (Shanghai, China), and divided into the sh‐NC group (injected with PaCa‐2 cells expressing control shRNA) and sh‐circ_0036627 group (injected with PaCa‐2 cells expressing sh‐circ_0036627) (*n* = 6 animals in each group). Circ_0036627‐silenced PaCa‐2 cells or control cells (2 × 10^6^ cells per animal) were suspended in 100 μL of PBS and subcutaneously injected into the left flanks of nude mice. Tumour size was measured using a calliper and tumour volume was quantified using the following formula (0.5 × L × W^2^), where L represents length and W indicates width. The mice were euthanized by cervical dislocation on Day 22 after injection, and tumour tissues were surgically collected for further analysis.

### 2.10 Western blot

RIPA lysis buffer (Beyotime, Shanghai, China) was applied to lyse the cells for protein isolation and the protein concentration was determined by a BCA protein assay kit (Beyotime, Beijing, China). In 10% SDS‐PAGE gels, equal quantities of protein were separated and then transferred to PVDF membranes (Millipore, Darmstadt, Germany). The membranes were blocked for 2 h with 5% nonfat dry milk before being treated with primary antibodies (anti‐S100A16, 1:1000, Cell Signaling Technology, Danvers, MA, USA; anti‐β‐actin, 1:5000, Cell Signaling Technology; anti‐PI3K antibody, 1:1000, Abcam, Cambridge, UK; anti‐phospho PI3K antibody, 1:1000, Abcam; anti‐AKT antibody, 1:1000, Abcam; anti‐phospho AKT antibody, 1: 1000, Abcam) overnight at 4°C. The membranes were then treated with HRP‐conjugated secondary antibody for 2 h. The protein bands were developed with an enhanced chemiluminescence kit (Beyotime, Shanghai, China).

### 2.11 Luciferase reporter assay

The predicted wildtype binding sites (WT) and the mutated sequences (MUT) were synthesized and subcloned into the pGL3‐basic vector (Promega, Madison, WI, USA) by RiboBio (Guangzhou, China). PC cells were seeded in a 12‐well plate at a density of 5 × 10^5^ cells per well and transfected with WT or MUT reporter in combination of miR‐145 mimic or control mimic (miR‐NC). Dual‐Luciferase Reporter Assay System kit (Promega, Madison, WI, USA) was used to detect the relative activity of firefly and Renilla luciferase.

### 2.12 Colony formation assay

Cells with indicated treatment were trypsinized and resuspended in culture medium, and seeded into a 6‐well plate (2000 cells/well). The cells were cultivated at 37°C and the medium was changed every 3 days. After 14 days, cells were fixed with 4% paraformaldehyde at room temperature for 10 min and stained with 0.5% crystal violet (Beyotime, Shanghai, China) for 20 min. The number of colonies was counted under a phase‐contrast microscope.

### 2.13 Statistical analysis

All the data were displayed as the mean ± standard deviation (SD). Student's *t*‐test (two‐tailed) was used to determine the significance between the two groups. One‐way anova was employed to evaluate the differences among multiple groups, with Tukey's test as the post‐hoc analysis for pairwise comparisons. *p* < 0.05 was used as the probability threshold for statistical significance. Significant *p*‐values were displayed as follows: *p < 0.05, **p < 0.01, ***p < 0.001.

## 3 RESULTS

### 3.1 Elevated circ_0036627 expression in PC patients and its relationship to PC prognosis

**FIGURE 1:**
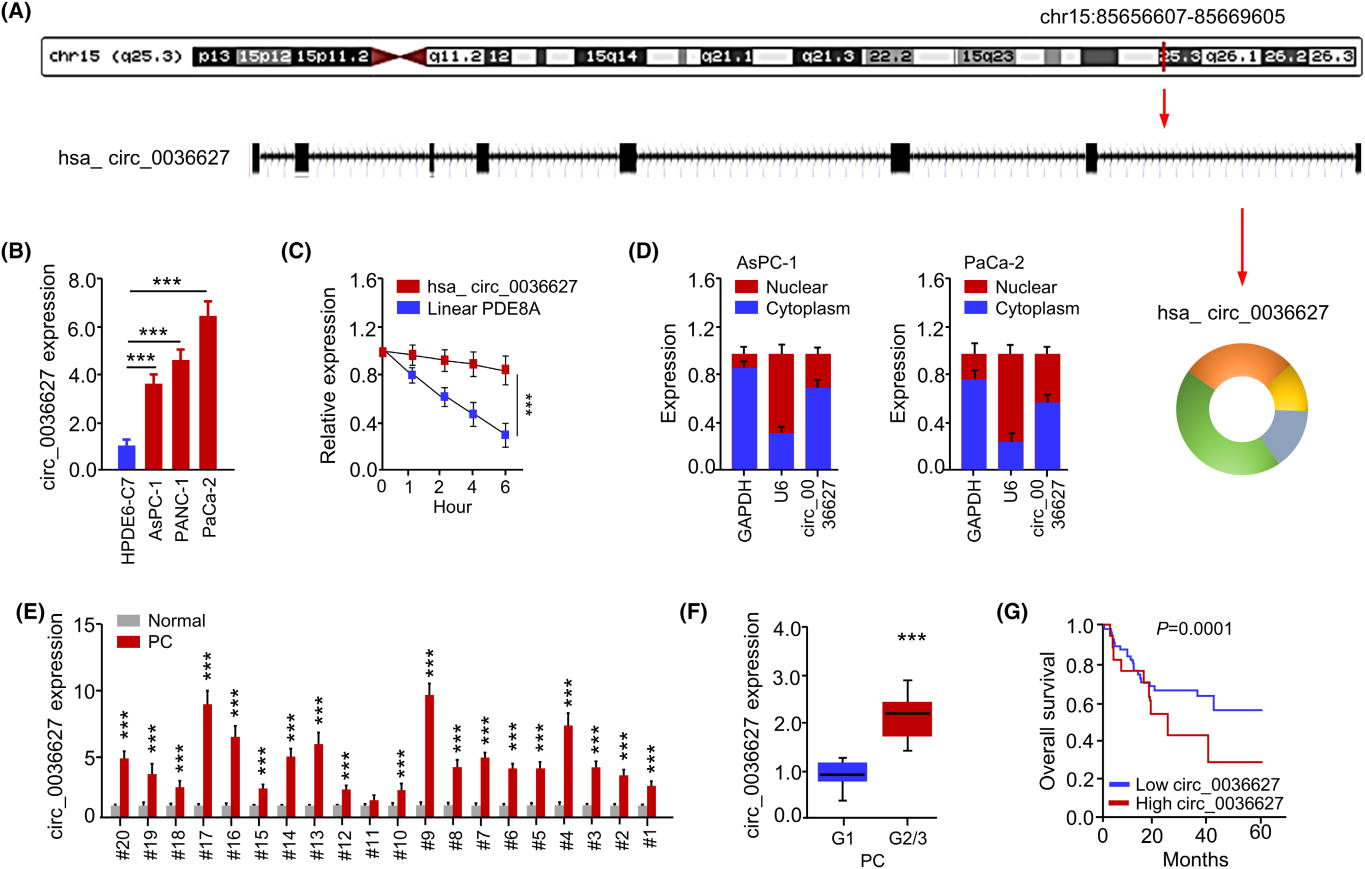
Elevated circ_0036627 expression in PC patients and its relationship to PC prognosis. (A) The schematics of genomic origin of circ_0036627 sequence. (B) qRT‐PCR was used to determine the relative levels of circ_0036627 in PC cell lines (AsPC‐1, PANC‐1 and PaCa‐2) and immortalized pancreatic duct epithelial cell line (HPDE6‐C7). (C) In PaCa‐2 cells treated with Actinomycin D, the relative abundance of circ_0036627 and linear PDE8A was detected via qRT‐PCR. (D) qRT‐PCR was used to quantify the subcellular distribution of circ_0036627 in the nuclear and cytoplasmic compartments. U6 and GAPDH were used as the nuclear and cytoplasmic control respectively. (E) qRT‐PCR was used to determine the relative levels of circ_0036627 in 20 pairs of PC tumour samples and adjacent normal tissues. (F) The analysis of circ_0036627 expression levels in PC patients diagnosed with low (G1) or high‐grade (G2/3) tumours. (G) Kaplan–Meier survival curve of PC patients with high (*n* = 30) or low circ_0036627 expression (*n* = 30). ***p < 0.001.

By analysing human genome via CircInteractome database, we found that circ_0036627 (chr15:85656607‐85669605) was composed of eight exons of the PDE8A gene (Figure 1A). qRT‐PCR analysis showed that circ_0036627 was highly expressed in PC cell lines (including AsPC‐1, PANC‐1 and PaCa‐2) compared to the immortalized pancreatic duct epithelial cell line (HPDE6‐C7) (Figure 1B). Thus, the PC cell lines AsPC‐1 and PaCa‐2 were chosen for further investigations because circ_0036627 was expressed at different levels in these two cell lines. The stability of circ_0036627 and the linear form of PDE8A mRNA in PaCa‐2 cells were examined with Actinomycin D treatment (Figure 1D). circ_0036627 remained stable while linear PDE8A mRNA showed a gradual decrease after Actinomycin D treatment (Figure 1E). The localization of circ_0036627 was analysed in AsPC‐1 and PaCa‐2 cells after the nucleo‐cytoplasmic separation. There was a relatively higher abundance of circ_0036627 in the cytoplasmic fraction (Figure 1D). We also examined the expression profiles of circ_0036627 in 20 pairs of PC tissue samples and the matched normal tissues. There was a significant upregulation of circ_0036627 in the majority of PC tumour samples (Figure 1E). The relative levels of circ_0036627 were also higher in the PC tissues of higher tumour grade (G2/3 compared to G1) (Figure 1F). PC patients with high circ_0036627 expression level (*n* = 30) exhibited a poorer overall survival compared to the low‐expression group (*n* = 30), as demonstrated by Kaplan–Meier survival analysis (Figure 1G). Therefore, circ_0036627 overexpression is associated with a poor prognosis in PC patients.

### 3.2 The knockdown of circ_0036627 impairs the aggressiveness of PC cells and promotes the sensitivity to GEM

**FIGURE 2:**
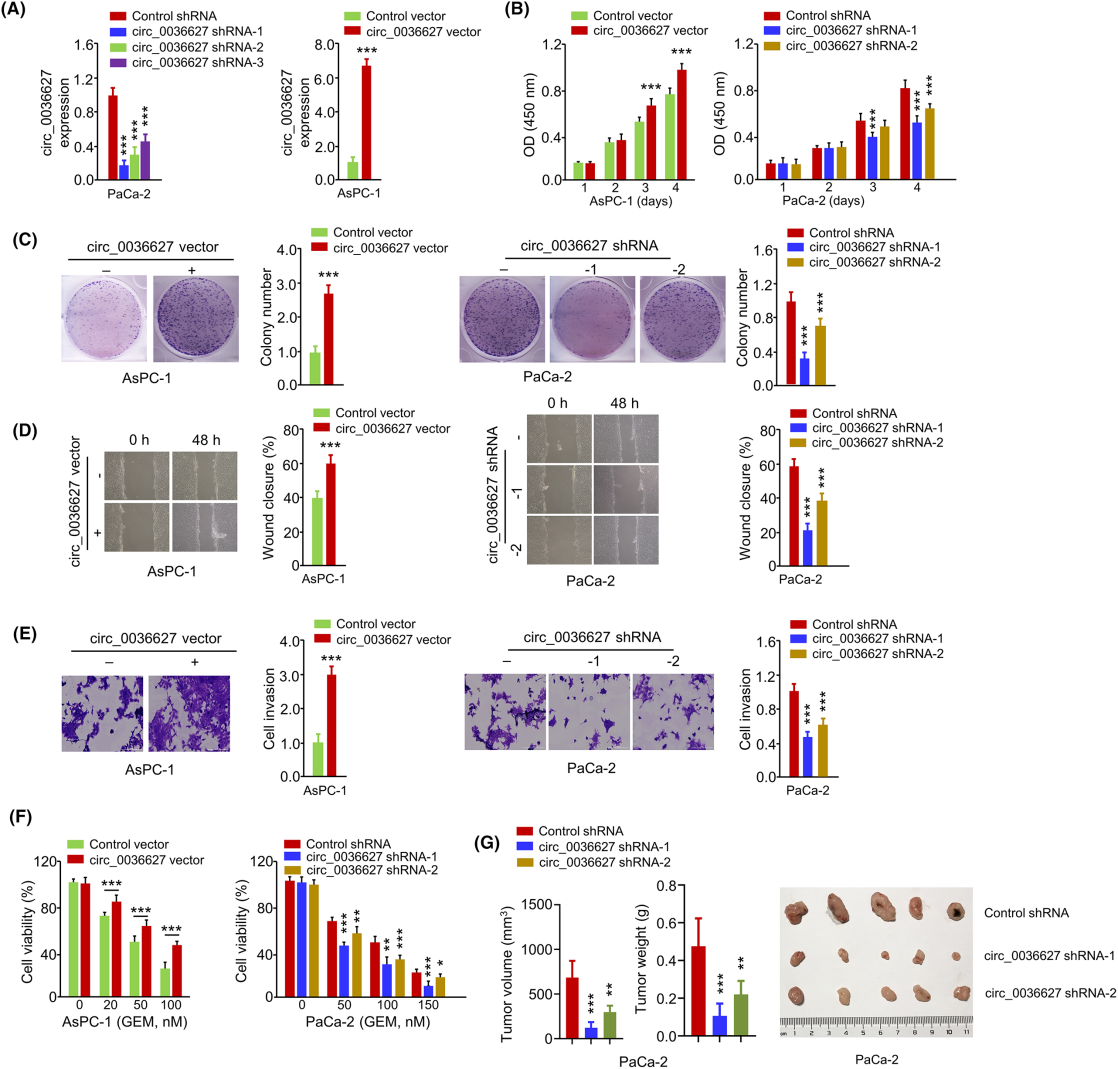
The knockdown of circ_0036627 impairs the aggressiveness of PC cells and promotes sensitivity to GEM. (A) circ_0036627 was silenced by shRNA (#1–3) or overexpressed by plasmid transfection in PaCa‐2 and AsPC‐1 cells, respectively. qRT‐PCR was used to detect circ_0036627 expression levels. shRNA #1 and #2 were used for the knockdown experiments. (B) CCK‐8 proliferation assay, (C) colony formation assay, (D) Wound‐healing assay, (E) transwell invasion assay and (F) GEM sensitivity assessment. (G) Nude mice were injected with PaCa‐2 cells with stable expression of circ_0036627 shRNA or shRNA (*n* = 6 animals in each group). The tumour volume and tumour weight were measured on day 22 after cell injection. ***p < 0.001.

In order to investigate the functional roles of circ_0036627, circ_0036627 was silenced by shRNA or overexpressed by plasmid transfection in PaCa‐2 and AsPC‐1 cells, respectively (Figure 2A). The cells transfected with circ_0036627, shRNA‐1 and shRNA‐2 showed high knockdown efficiency, which were selected for the subsequent investigations (Figure 2A). Functional experiments of CCK‐8 proliferation assay, colony formation assay, wound‐healing assay, invasion assay and cell viability assay revealed that silencing of circ_0036627 suppressed cell growth, migration, invasion and promoted the sensitivity to GEM in PaCa2 cells (Figure 2B–F). On the contrary, circ_0036627 overexpression showed opposite effects in AsPC‐1 cells (Figure 2B–F). When nude mice were injected with PaCa‐2 cells with stable expression of circ_0036627 shRNA or control shRNA, the knockdown of circ_0036627 significantly suppressed tumour growth (tumour volume and weight) in nude mice (Figure 2G). These results suggest that circ_0036627 overexpression promotes the malignant properties and GEM resistance of PC cells.

### 3.3 Circ_0036627 interacts with miR‐145

**FIGURE 3:**
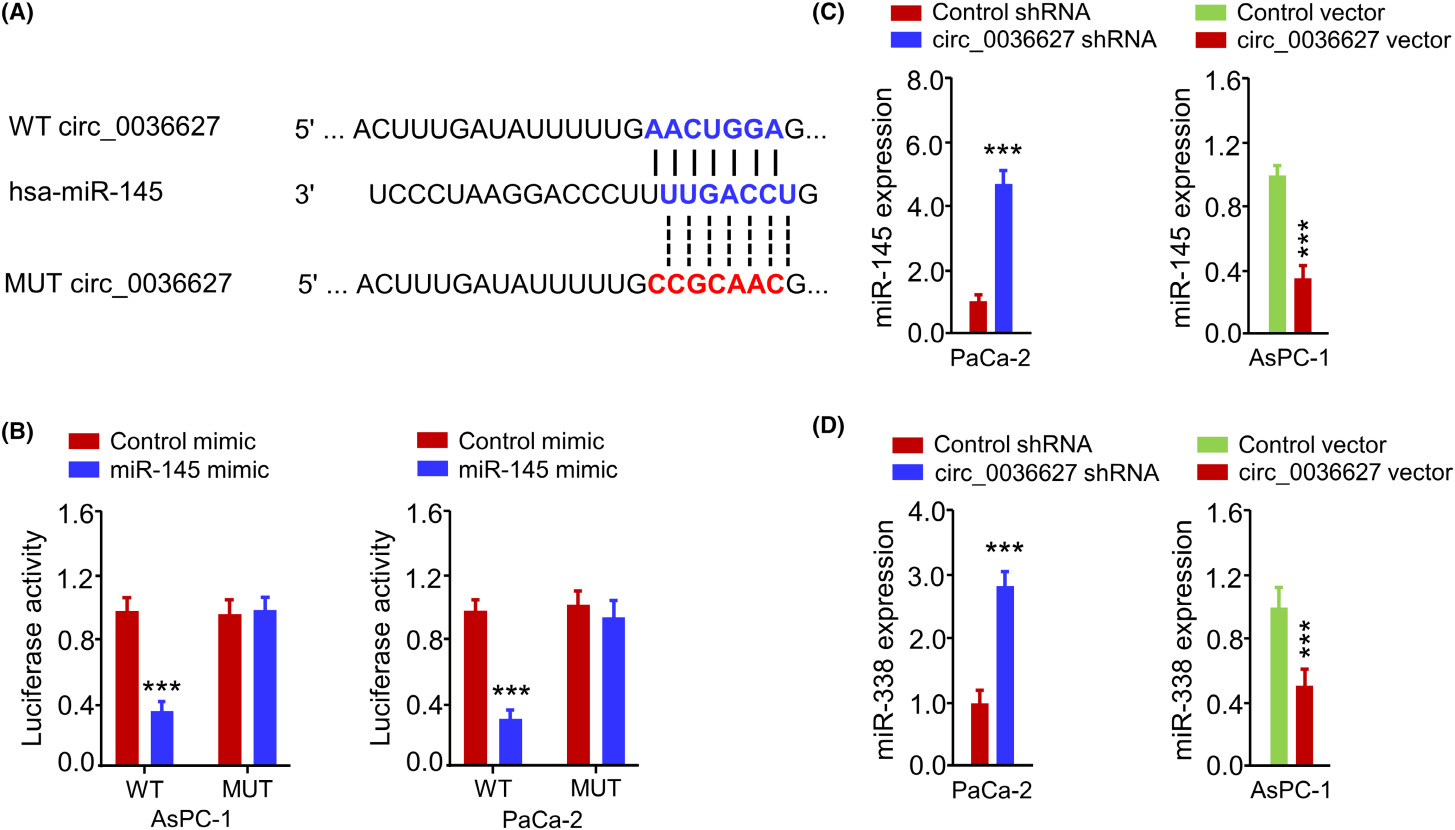
Circ_0036627 interacts with miR‐145. (A) Wild‐type (WT) and mutant (MUT) circ_0036627/miR‐145 interaction sequences predicted by TargetScan online tool. (B) Dual‐luciferase reporter assay in PC cells using circ_0036627 WT or MUT reporter in the presence of miR‐145 mimic or miR‐NC. (C, D) The levels of miR‐145 (C) or miR‐338 (D) in PC cells were detected by qRT‐PCR after the transfection of circ_0036627 shRNAs (or control shRNA), or circ_0036627 expression vector (or control vector). ***p < 0.001.

We also determined the mechanism through which circ_0036627 modulates cellular features in PC cells. Based on the TargetScan database, multiple miRNAs were predicted to be potential targets of circ_0036627 (Figure S1) and the expression levels of these miRNAs were detected in PC cells with circ_0036627 shRNA or control shRNA. The data showed that only miR‐145 was significantly upregulated upon circ_0036627 knockdown, indicating that circ_0036627 may regulate miR‐145 expression in PC cells (Figure S1). There were potential interaction binding sequences between miR‐145 and circ_0036627 (Figure 3A). Since miR‐145 acts as a tumour suppressor to negatively regulate several oncogenes in PC cells,^12^, ^13^, ^14^, ^15^ circ_0036627 functions as a miR‐145 ‘sponge’ to impinge on the malignancy of PC cells. Through the dual luciferase reporter assay, miR‐145 overexpression suppressed the activity of wild‐type (WT) circ_0036627 reporter in AsPC‐1 and PaCa‐2 cells (Figure 3B). When the miR‐145 target sites were mutated (MUT reporter), the effect of miR‐145 overexpression was abolished (Figure 3B). In PaCa‐2 cells, circ_0036627 silencing elevated the expression level of miR‐145, while circ_0036627 overexpression repressed miR‐145 level (Figure 3C). miR‐338 was previously reported to interact with circ_0036627 in PCs.^11^ We also found that miR‐338 expression was increased by circ_0036627 knockdown and decreased by circ_0036627 overexpression (Figure 3D). These findings indicate that circ_0036627 binds to miR‐145 (and other miRNAs) to reduce their expression.

### 3.4 miR‐145 acts a tumour‐suppressive miRNA in PC cells

**FIGURE 4:**
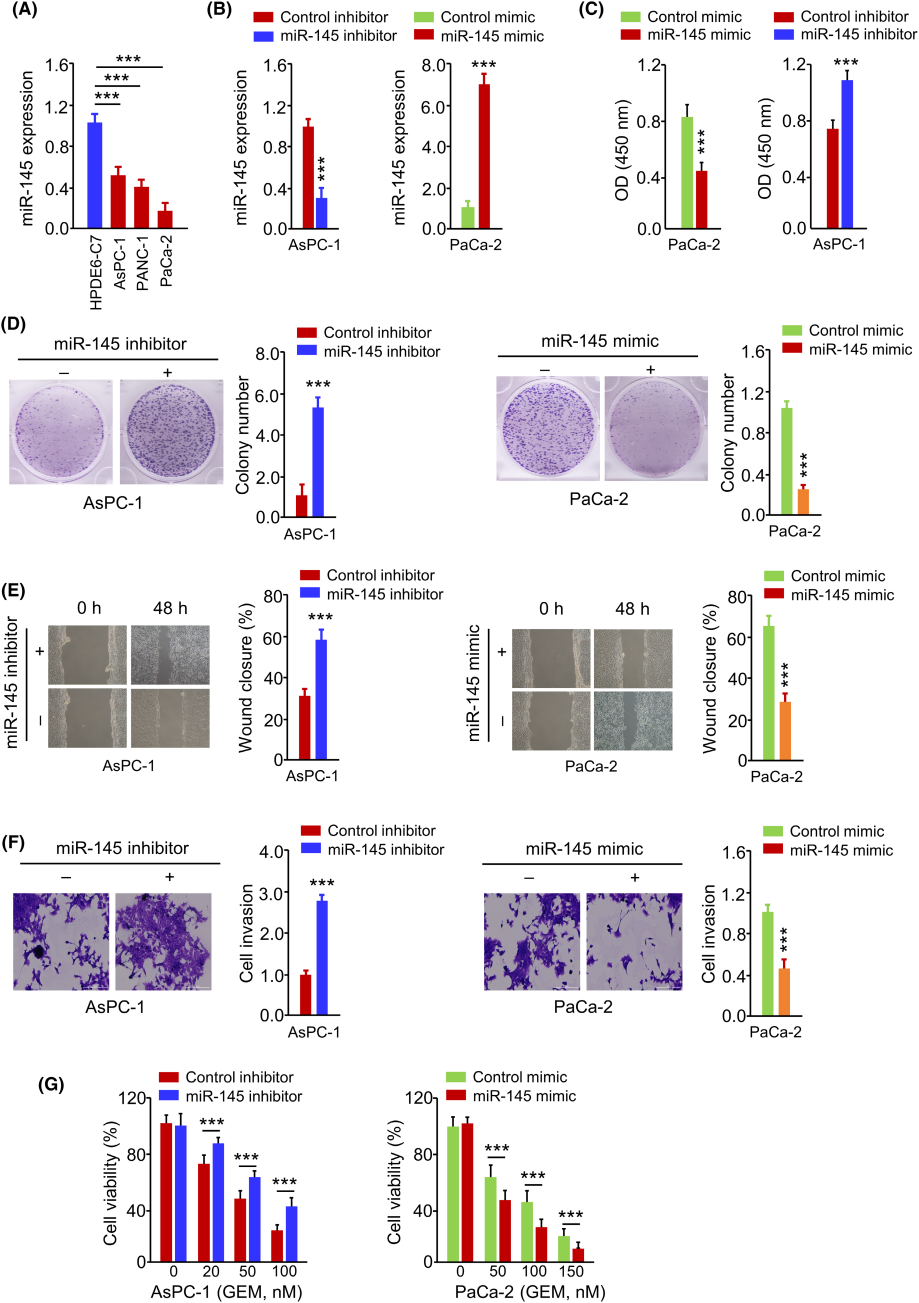
miR‐145 acts as a tumour‐suppressive miRNA in PC cells. (A) qRT‐PCR analysis of miR‐145 expression in PC cell lines (AsPC‐1, PANC‐1 and PaCa‐2) and immortalized pancreatic duct epithelial cell line (HPDE6‐C7). (B) Analysis of miR‐145 expression levels in PC cells transfected with miR‐145 mimic or miR‐145 inhibitor. (C) CCK‐8 proliferation assay, (D) colony formation assay, (E) wound‐healing assay, (F) transwell invasion assay and (G) GEM sensitivity assessment in PC cells transfected with miR‐145 mimic or miR‐145 inhibitor. ***p < 0.001.

We then analysed the expression and function of miR‐145 in PC cells. miR‐145 was found to be downregulated in PC cells compared to the normal cells (Figure 4A). To modulate miR‐145 levels, we transfected miR‐145 mimic in PaCa‐2 cells or miR‐145 inhibitor in AsPC‐1 cells, which caused the overexpression or downregulation of miR‐145 respectively (Figure 4B). miR‐145 mimic dramatically suppressed cell growth, colony formation, migration, invasion and GEM resistance in PaCa‐2 cells (Figure 4C–G). The inhibition of miR‐145 expression promoted the growth, motility, invasion and GEM resistance in AsPC‐1 cells (Figure 4C–G). These data demonstrate that miR‐145 acts as a tumour suppressor to attenuate the aggressiveness and GEM resistance in PC cells.

### 3.5 miR‐145 negatively regulates S100A16 expression

**FIGURE 5:**
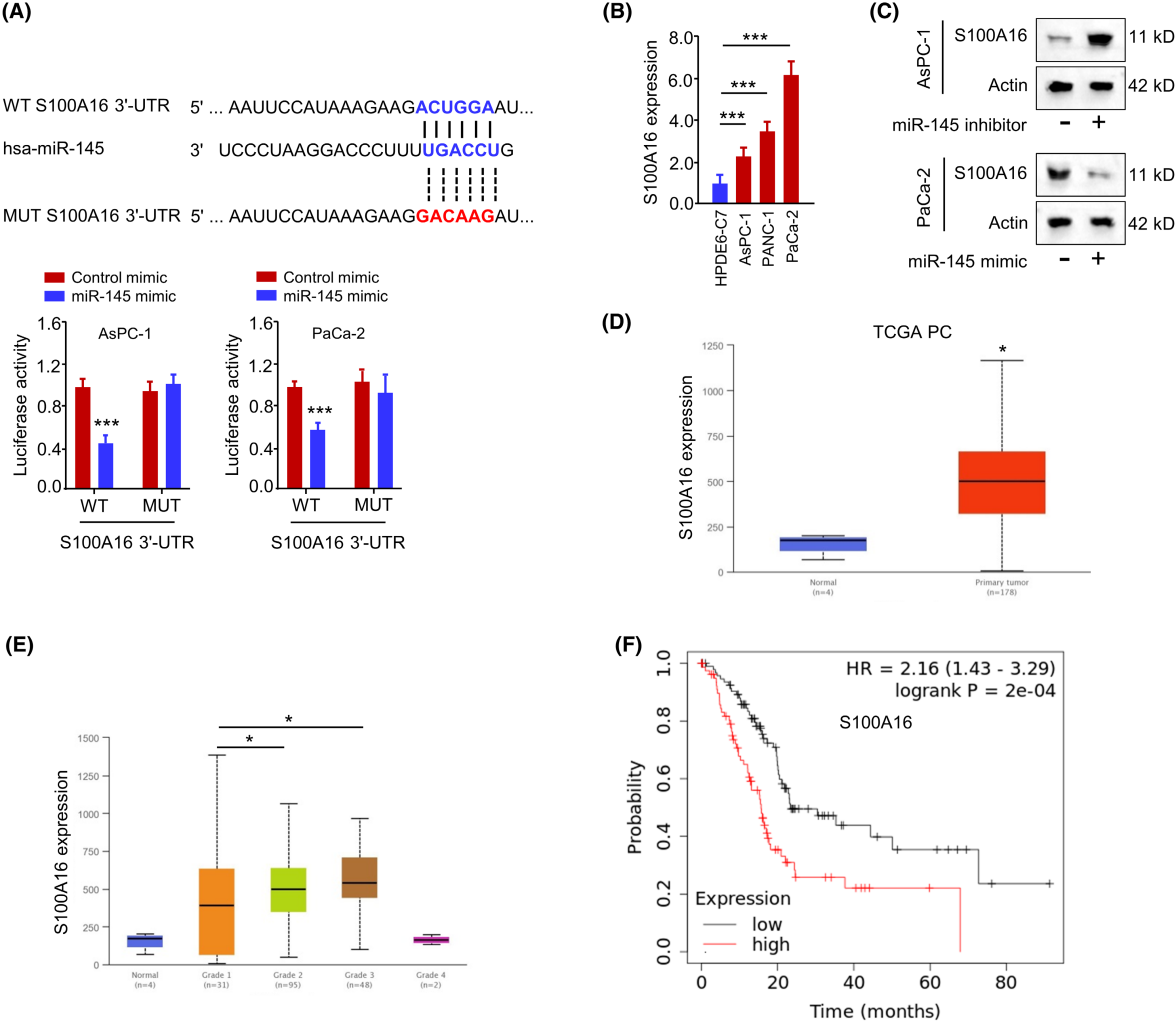
miR‐145 negatively regulates S100A16 expression. (A) The predicted miR‐145 WT binding sites or MUT sequences with the 3'‐UTR of S100A16 mRNA by TargetScan online tool, and the interaction verification by dual‐luciferase reporter assay. (B). S100A16 expression analysed by qRT‐PCR in PC cell lines (AsPC‐1, PANC‐1 and PaCa‐2) and immortalized pancreatic duct epithelial cell line (HPDE6‐C7). (C) S100A16 protein levels in PC cells transfected with miR‐145 mimic or miR‐145 inhibitor were examined by Western blot. (D) S100A16 mRNA levels in PC tissues and normal tissues of TCGA PC patient cohort analysed through UALCAN database. (E) S100A16 levels in the samples of different tumour grades in TCGA PC patient cohort (UALCAN database). (F) The Kaplan–Meier survival curve analysis in TCGA PC patient cohort (UALCAN database) with high or low S100A16 expression. ***p < 0.001.

Furthermore, we predicted the mRNA targets of miR‐145 using TargetScan online tools. Among the predicted mRNA targets, S100A16 mRNA showed significant upregulation in PC cells after the transfection of miR‐145 inhibitor, indicating that miR‐145 is a negative regulator of S100A16 (Figure S2). TargetScan tool predicted that miR‐145 shared potential complementary sequences with the S100A16 mRNA 3'‐UTR sequence. The luciferase activity of WT S100A16 3'‐UTR reporter, but not MUT S100A16 3'‐UTR reporter, was suppressed by miR‐145 mimic (Figure 5A). S100A16 mRNA level was significantly increased in PC tumour tissues when compared to normal tissues (Figure 5B). miR‐145 overexpression decreased the protein level of ST100A16, while miR‐145 inhibitor increased the expression of S100A16 protein (Figure 5C). Through the profiling of PC patient cohort dataset in the database UALCAN, S100A16 expression was also significantly elevated in PC tumour tissues when compared to normal tissues (Figure 5D) and the relative levels of S100A16 were higher in Grade 2 and 3 tumour samples when compared to Grade 1 samples (Figure 5E). A high level of S100A16 expression was also correlated with a poorer prognosis in PC patients (Figure 5F). These findings indicate that miR‐145 negatively regulates S100A16 in PC cells and elevated S100A16 expression is linked with a poor survival in PC patients.

### 3.6 S100A16 overexpression enhances the aggressiveness and resistance to GEM in PC cells

**FIGURE 6:**
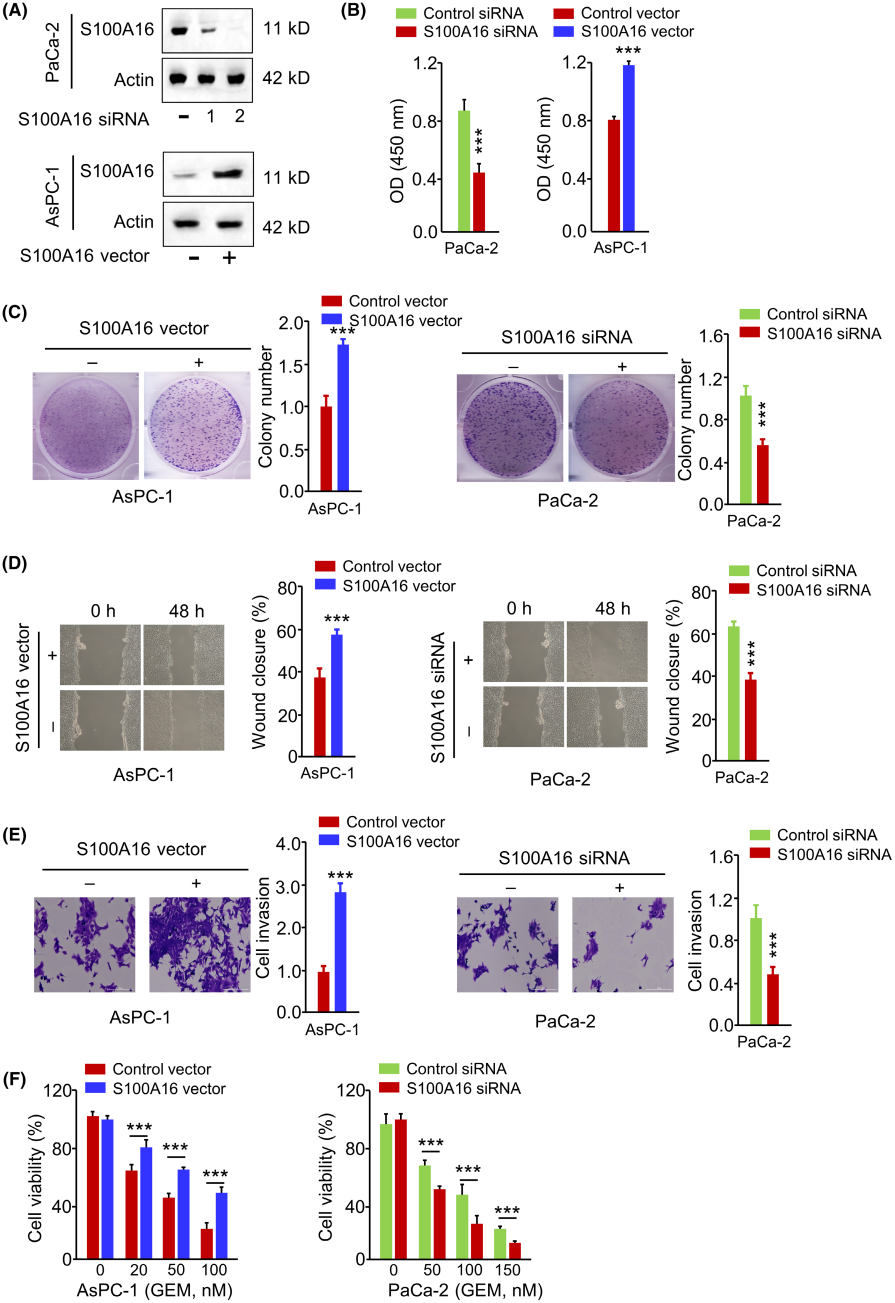
S100A16 overexpression enhances the aggressiveness and resistance to GEM in PC cells. (A) S100A16 protein levels in PC cells after the transfection with S100A16 expression vector or S100A16 siRNA. (B) CCK‐8 proliferation assay, (C) colony formation assay, (D) wound‐healing assay, (E) transwell invasion assay and (F) GEM sensitivity assessment in PC cells after the transfection with S100A16 vector or S100A16 siRNA. ***p < 0.001.

**FIGURE 7:**
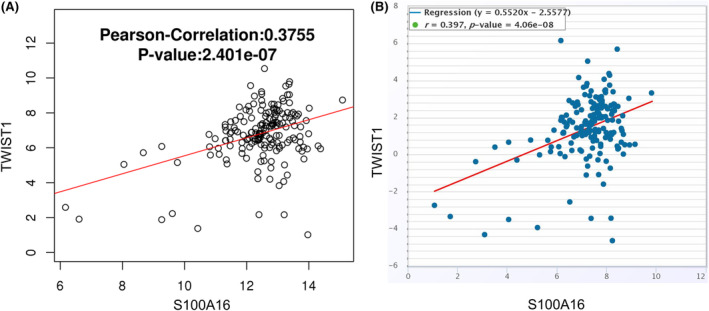
The relationship between S100A16 and TWIST1 expression. (A) The LinkedOmics database and (B) the ENCORI database were used to explore the correlation between S100A16 and TWIST1 levels in the TCGA cohort of PC samples.

To demonstrate the functional role of S100A16 in PC cells, the overexpression or knockdown of S100A16 was achieved by transfecting PC cells with S100A16 siRNA‐2 or S100A16 expression vector (Figure 6A). S100A16 silencing inhibited PaCa‐2 cell growth and colony formation (Figure 6B,C), suppressed the migration and invasion in Paca‐2 cells (Figure 6D,E) and promoted the cellular sensitivity towards GEM treatment (Figure 6F). In contrast, the overexpression of S100A16 in AsPC‐1 cells promoted cell growth, migration, invasion and GEM resistance (Figure 6B–E). To reveal the possible mechanisms by which S100A16 mediates GEM resistance and the aggressiveness of PC cells, we assessed the correlation between S100A16 and a known oncogene TWIST1 in TCGA cohort of PC samples using two online databases (LinkedOmics and ENCORI). There was a significant positive association between S100A16 and TWIST1 expression levels in PC tissues (Figure 7). These findings suggest that S100A16 overexpression enhances the aggressiveness and resistance to GEM in PC cells, and correlated with TWIST1 expression in PC samples.

### 3.7 circ_0036627 regulates cell proliferation, invasion and GEM resistance via suppressing miR‐145

**FIGURE 8:**
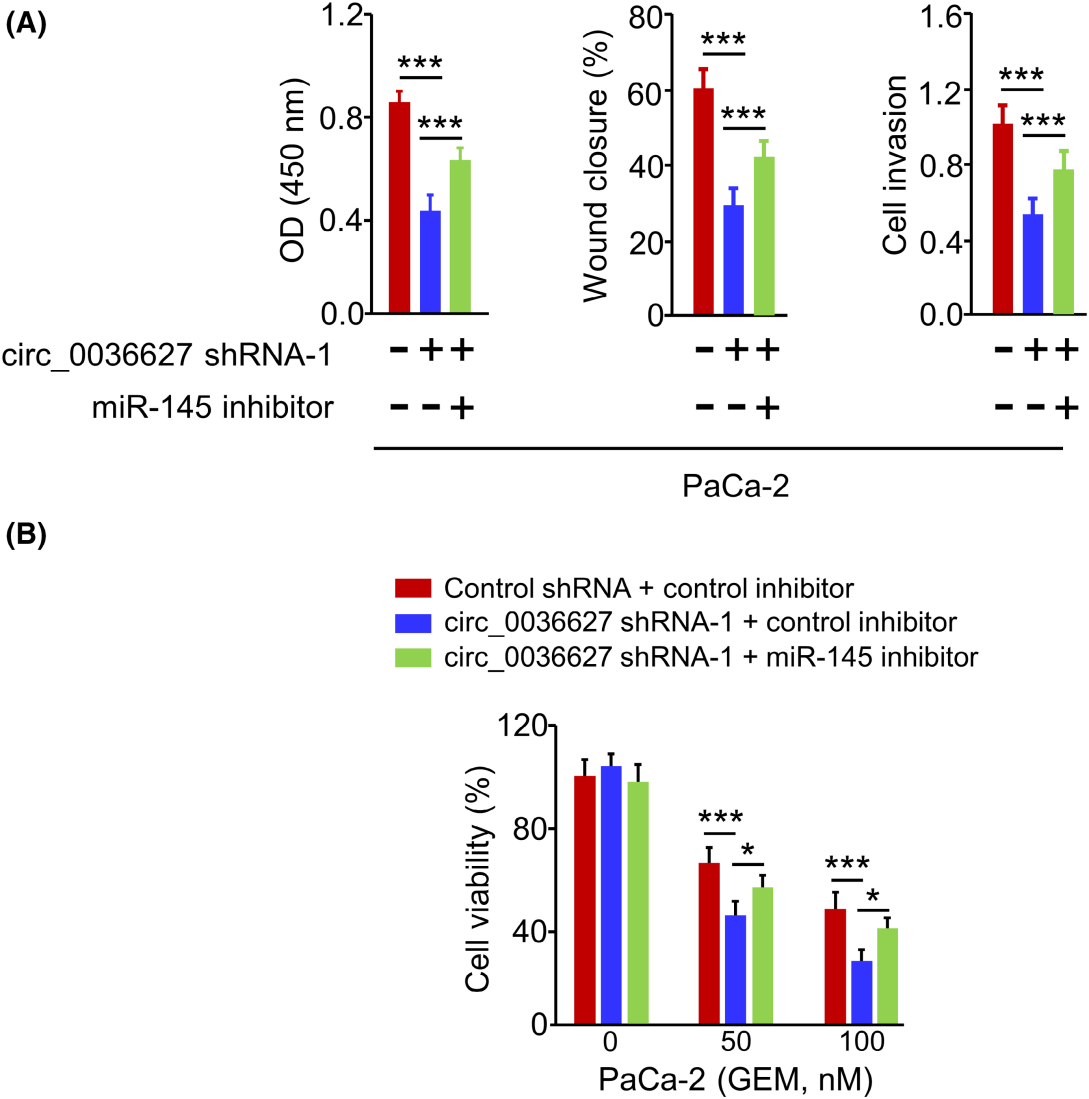
circ_0036627 regulates cell proliferation, invasion and GEM resistance via suppressing miR‐145. (A) Cell proliferation, migration and invasion abilities and (B) GEM sensitivity were analysed in PaCa‐2 cells transfected with control shRNA+control inhibitor, circ_0036627 shRNA+control inhibitor, or circ_0036627 shRNA and miR‐145 inhibitor. ***p < 0.001.

According to our findings, circ_0036627 sponges miR‐145, whereas miR‐145 negatively impacts S100A16. Therefore, we investigated whether circ_0036627 impinges on PC cell proliferation, invasion and GEM resistance via miR‐145. PaCa‐2 cells were transfected with circ_0036627 shRNA or/and miR‐145 inhibitor were subjected to functional assay. Reduced expression of circ_0036627 suppressed the proliferation, migration and invasion of PaCa‐2 cells and miR‐145 inhibition attenuated the effects of circ_0036627 knockdown (Figure 8A). circ_0036627 silencing rendered PaCa‐2 cells more sensitive to GEM treatment, while miR‐145 inhibitor restored the resistance to GEM (Figure 8B). These findings indicate that circ_0036627 promotes aggressiveness and GEM resistance in PC cells by sponging miR‐145.

### 3.8 S100A16 regulates PI3K/AKT pathway in PC cells

**FIGURE 9:**
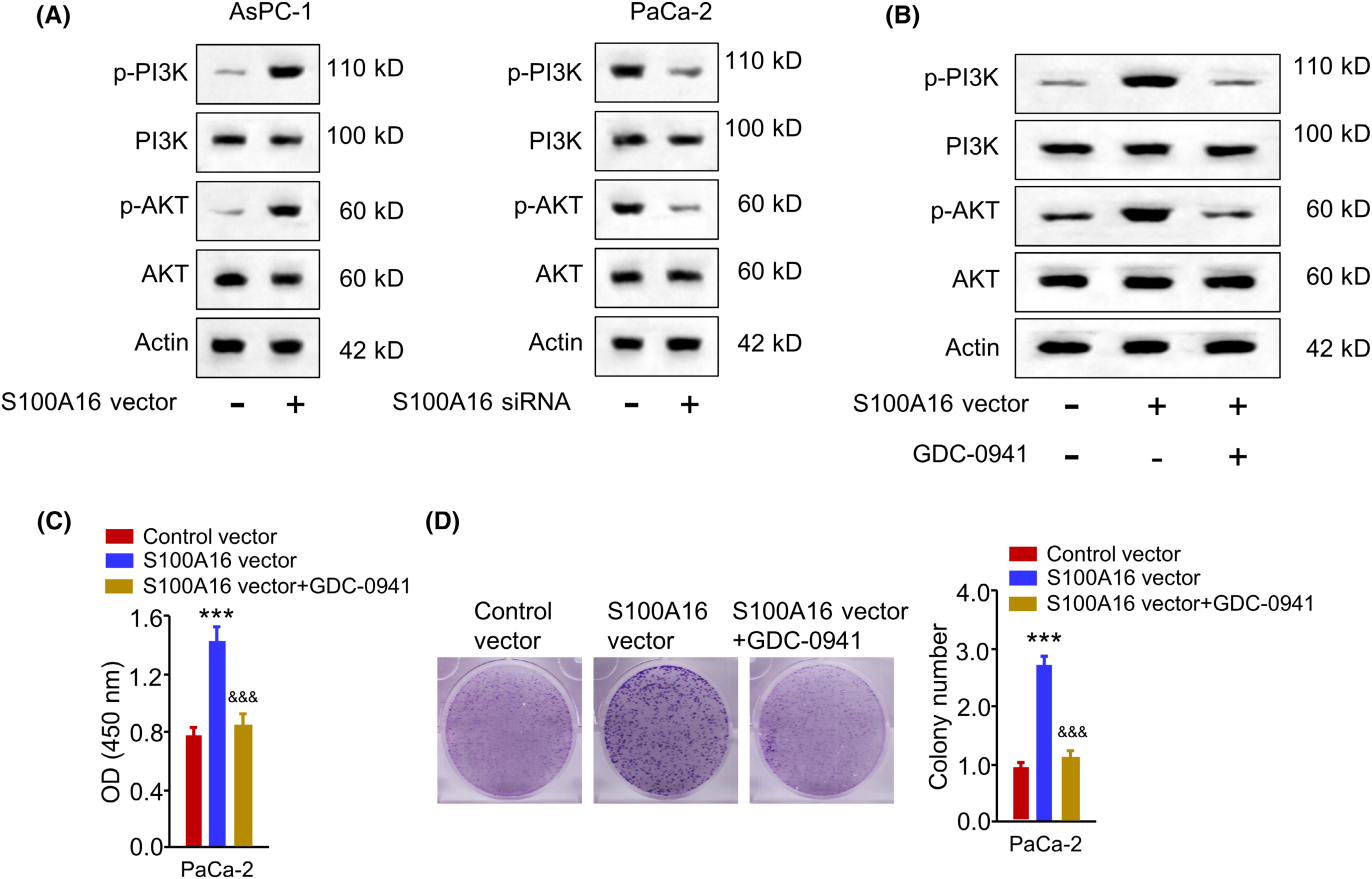
S100A16 regulates PI3K/AKT signalling pathway in PC cells. (A) PC cells were transfected with S100A16 overexpression plasmid or siRNA, and the phosphorylation levels of PI3K and AKT were examined by Western blot. (B–D) PC cells were transfected with an S100A16 expression vector and treated with PI3K inhibitor (GDC‐0941, 2 μM). (B) The phosphorylation levels of PI3K and AKT were examined by Western blot. (C) GEM sensitivity assessment. (D) Colony formation assay. ***p < 0.001 versus control vector; ^###^ *p* < 0.001 versus S100A16 vector.

Next, we wonder whether S100A16 modulates the malignancy of PC cells through PI3K/AKT signalling since a previous study showed that S100A16 regulates the malignancy of HeLa cell through the PI3K/AKT signalling pathway.^16^ S100A16 overexpression promoted the phosphorylation of PI3K and AKT, while S100A16 silencing showed an opposite effect (Figure 9A). Further, PC cells were transfected with the S100A16 expression vector and treated with PI3K inhibitor (GDC‐0941). GDC‐0941 suppressed the activation of PI3K/AKT signalling upon S100A16 overexpression (Figure 9B). Further, GDC‐0941 suppressed the resistance to GEM in PC cells upon S100A16 overexpression (Figure 9C). GDC‐0941 suppressed the colony formation ability in PC cells after S100A16 overexpression (Figure 9D). These data suggest that S100A16 activates the PI3K/AKT pathway to modulate the malignancy and GEM resistance in PC cells.

## 4 DISCUSSION

At the time of diagnosis, a considerable number of PC patients are diagnosed with local invasion or metastasis, and these patients are conventionally treated with chemotherapy or radiation treatment.^1^, ^2^, ^3^, ^4^ Even though GEM‐based chemotherapy can improve the survival of PC patients, its efficacy is compromised due to the presence of drug resistance.^1^, ^2^, ^3^, ^4^ Most human cancers have been associated with altered miRNA and circRNA expression.^17^ CircRNAs and miRNAs can function as oncogenes or tumour suppressors, impinging on cell proliferation, invasion and drug resistance.^18^ A previous study demonstrated that the expression of circ_0036627 was significantly elevated in highly invasive PC cells compared to PC cells with low invasive ability.^11^ According to this study, circ_0036627 acts as a sponge for miR‐338 to promote cell invasion.^11^ A large number of circRNAs have been found to be deregulated in different human malignancies through high‐throughput sequencing studies.^19^, ^20^, ^21^ Further, a growing number of circRNAs have been demonstrated to regulate the pathophysiological progression of PC.^22^ However, the potential role of circ_0036627 in the development of chemoresistance in PC cells is unknown.

In this study, we showed that circ_0036627 is highly expressed in PC tissue and cell lines, which is consistent with the previous study.^11^ Here, our findings suggest that circ_0036627 acts as an oncogenic factor in PC cells. Circ_0036627 upregulation was linked to a poorly differentiated state of PC cells. Overexpression of circ_0036627 augmented cell proliferation and promoted mobility in PC cells, and circ_0036627 silencing attenuated the tumorigenesis of PC cells in nude mice. Importantly, circ_0036627 overexpression conferred GEM chemoresistance in PC cells. Elevated circ_0036627 expression level was also associated with a poor prognosis in PC patients, indicating its potential as a prognostic indicator. Targeting circ_0036627 could serve as a strategy to suppress the malignancy and promote GEM sensitivity in PC treatment.

We explored the miRNA targets of circ_0036627 to reveal its underlying mechanisms. miR‐145 was identified as a potential target of circ_0036627, which was validated by the dual luciferase reporter assay. The overexpression of circ_0036627 reduced the miR‐145 level, and circ_0036627 silencing enhanced miR‐145 expression. According to the previous study, circ_0036627 also acts as a sponge for miR‐338 to promote the invasive feature of PC.^11^ Thus, circ_0036627 could serve as molecular sponges for several tumour suppressor miRNAs and, circ_0036627 may attenuate the miR‐145‐dependent process that confers GEM resistance.

miRNAs are important non‐coding RNAs involved in tumour progression and metastasis by altering the expression of their target genes.^8^ miR‐145 has been identified as a tumour suppressor in lung cancer, oesophageal squamous cell carcinoma, ovarian cancer, glioma, hepatocellular carcinoma, breast cancer, colorectal cancer, prostate cancer, bladder cancer, gastric cancer and PC.^12^, ^13^, ^14^, ^15^, ^23^ Different mechanisms contribute to drug resistance in cancers, including enhanced drug efflux, evasion of cell death, alterations in the drug targets, improved DNA repair and target gene amplification.^24^ miR‐145 was found to differentially regulate drug sensitivity in different malignancies. For example, overexpression of miR‐145 in breast cancer improved doxorubicin sensitivity by promoting intracellular drug accumulation via inhibiting MRP1.^25^ By directly blocking the PI3K/AKT signalling axis, miR‐145 also sensitizes oesophageal squamous cell carcinoma cells to cisplatin treatment by targeting MRP1 and Pgp expression.^26^ Another report showed that p70S6K1 overexpression was involved in GEM chemoresistance in PC cells.^27^ By directly targeting p70S6K1, miR‐145 enhanced the susceptibility of PC cells to GEM treatment.^27^ In line with these investigations, we showed that miR‐145 overexpression suppressed not only the malignant features of PC cells, but also attenuated GEM resistance. S100A16 was identified as a target gene for miR‐145 and the downregulation of S100A16 showed similar effects on PC cells as miR‐145 overexpression. These findings suggest that the miR‐145/S100A16 axis plays a critical role in the malignancy and GEM resistance in PC cells.

At least 21 members of the S100 family of proteins have been associated with different pathophysiological conditions, including cardiomyopathy, neurodegenerative diseases, inflammatory disorders and cancers.^28^ S100A16 has been widely reported to become dysregulated in a variety of human tumour tissues^29^ and its overexpression has been associated with the poor prognosis in colorectal cancer patients.^30^ Epithelial‐mesenchymal transition (EMT) and the development of cancer stem cell‐like phenotypes are involved in tumour progression, metastasis and drug resistance.^31^ S100A16 has been implicated in the maintenance of cancer stem‐like features in cervical carcinoma cells.^32^ The mRNA and protein expression of stem cell markers (Oct4 and Nanog) were reduced after S100A16 silencing and the spheroid formation ability of cervical cancer cells was considerably reduced upon S100A16 knockdown.^32^ S100A16 also promotes the metastasis of PC cells by inducing EMT, which is mediated by the increased TWIST1 expression and the activation of the STAT3 signalling pathway.^33^ The tumoricidal effect of GEM was improved when combined with S100A16 downregulation in PC cell lines (PANC‐1 and CFPAC‐1).^33^ Our results showed that S100A16 overexpression enhanced cell proliferation, migration, invasion and GEM sensitivity, while the silencing of circ_0036627 showed opposite effects. These data are consistent with the reported oncogenic role of S100A16.^31^, ^32^, ^33^ Notably, it is known that S100A16 promotes the progression and metastasis of PC by regulating FGF19‐mediated AKT and ERK1/2 pathways.^34^ Further, a previous study showed that S100A16 regulates the malignancy of HeLa cell through PI3K/AKT signalling pathway.^16^ Our data are consistent with these findings and indicate that S100A16 activates the PI3K/AKT pathway to modulate the malignancy and GEM resistance in PC cells. However, future efforts are required to elucidate the mechanism by which S100A16 overexpression activates PI3K/AKT signalling in PC cells.

## 5 CONCLUSIONS

**FIGURE 10:**
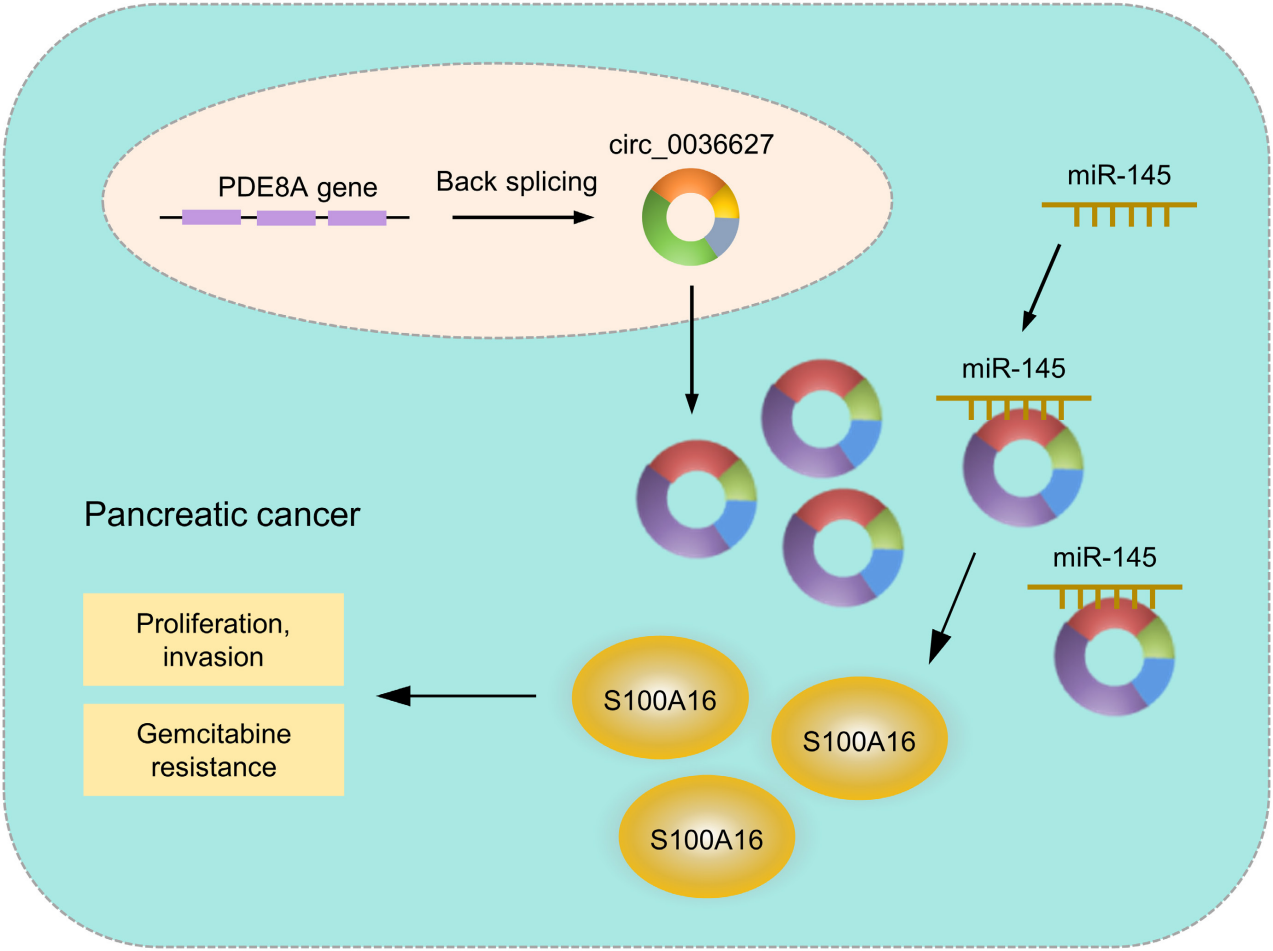
Schematics of the mechanism by which circ_0036627 regulates the malignancy and GEM resistance in PC cells by targeting miR‐145/S100A16 axis.

The current study discovered a new circRNA, circ_0036627, which is overexpressed in PC tissues and associated with overall survival in PC patients. Increased circ_0036627 expression promoted malignancy and GEM resistance in PC cells. Our findings also suggest that circ_0036627/miR‐145/S100A16 axis plays a crucial role in dictating the aggressiveness and GEM resistance in PC cells (Figure 10). Targeting circ_0036627 may serve as an intervention strategy to curb PC progression and improve the treatment outcome of GEM.

## AUTHOR CONTRIBUTIONS

**Shuo Yu:** Conceptualization (equal); investigation (equal); methodology (equal); writing – original draft (equal). **Min Wang:** Conceptualization (equal); investigation (equal). **Hang Zhang:** Investigation (equal); methodology (equal); writing – original draft (equal). **Xingjun Guo:** Methodology (equal); writing – original draft (equal). **Renyi Qin:** Conceptualization (equal); data curation (equal); investigation (equal); methodology (equal); writing – original draft (equal).

## FUNDING INFORMATION

No funding was used in this study.

## CONFLICT OF INTEREST STATEMENT

The authors declare no conflicts of interest.

## CONSENT

All cases provided the informed consent.

## Supporting information


Figure S1.

Figure S2.

## Data Availability

The data that support the findings of this study are available from the corresponding author upon reasonable request.
