# Supplementary figures and images for: Resistance to gemcitabine is mediated by the circ_0036627/miR‐145/S100A16 axis in pancreatic cancer
Source: J Cell Mol Med. 2024 Jun 24;28(12):e18444. doi: 10.1111/jcmm.18444 (PMC11196374; doi:10.1111/jcmm.18444)

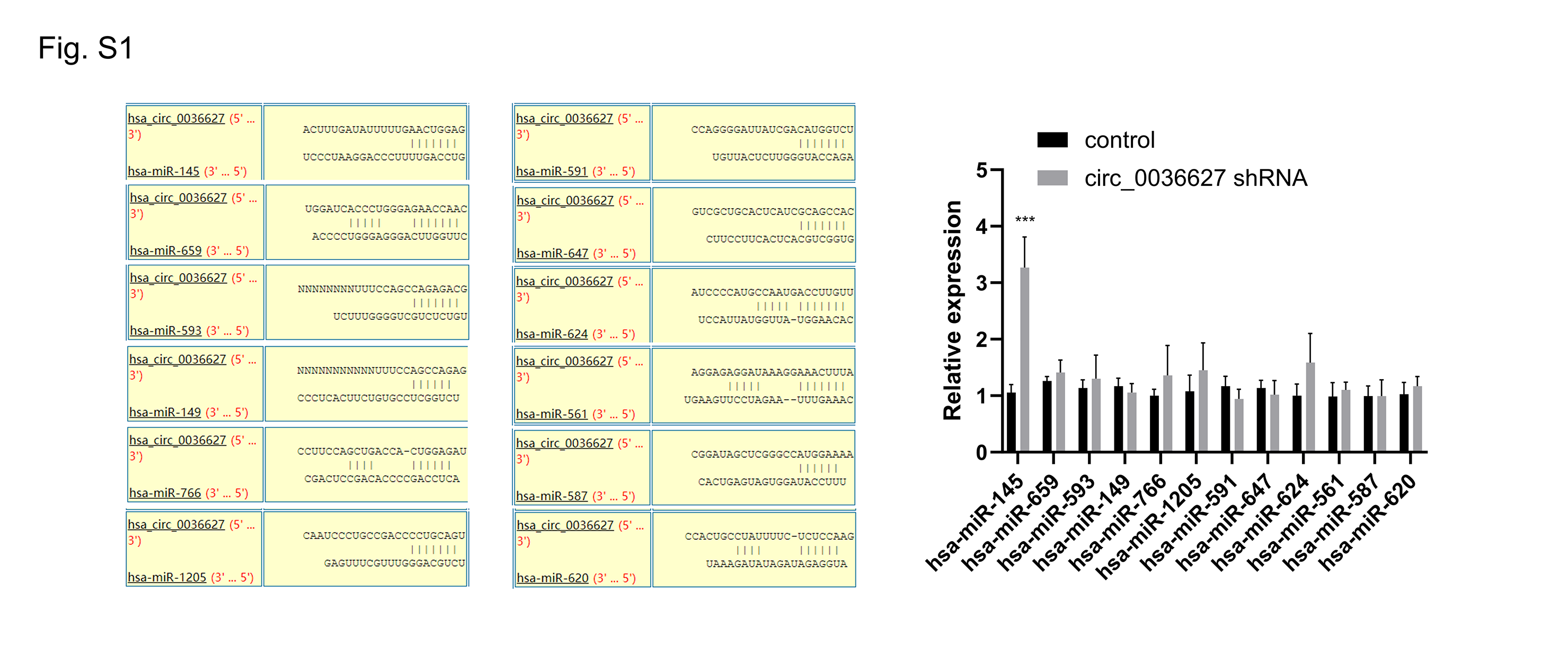

Supplement: Supplementary file 1 — Figure S1. [file JCMM-28-e18444-s002.tif]

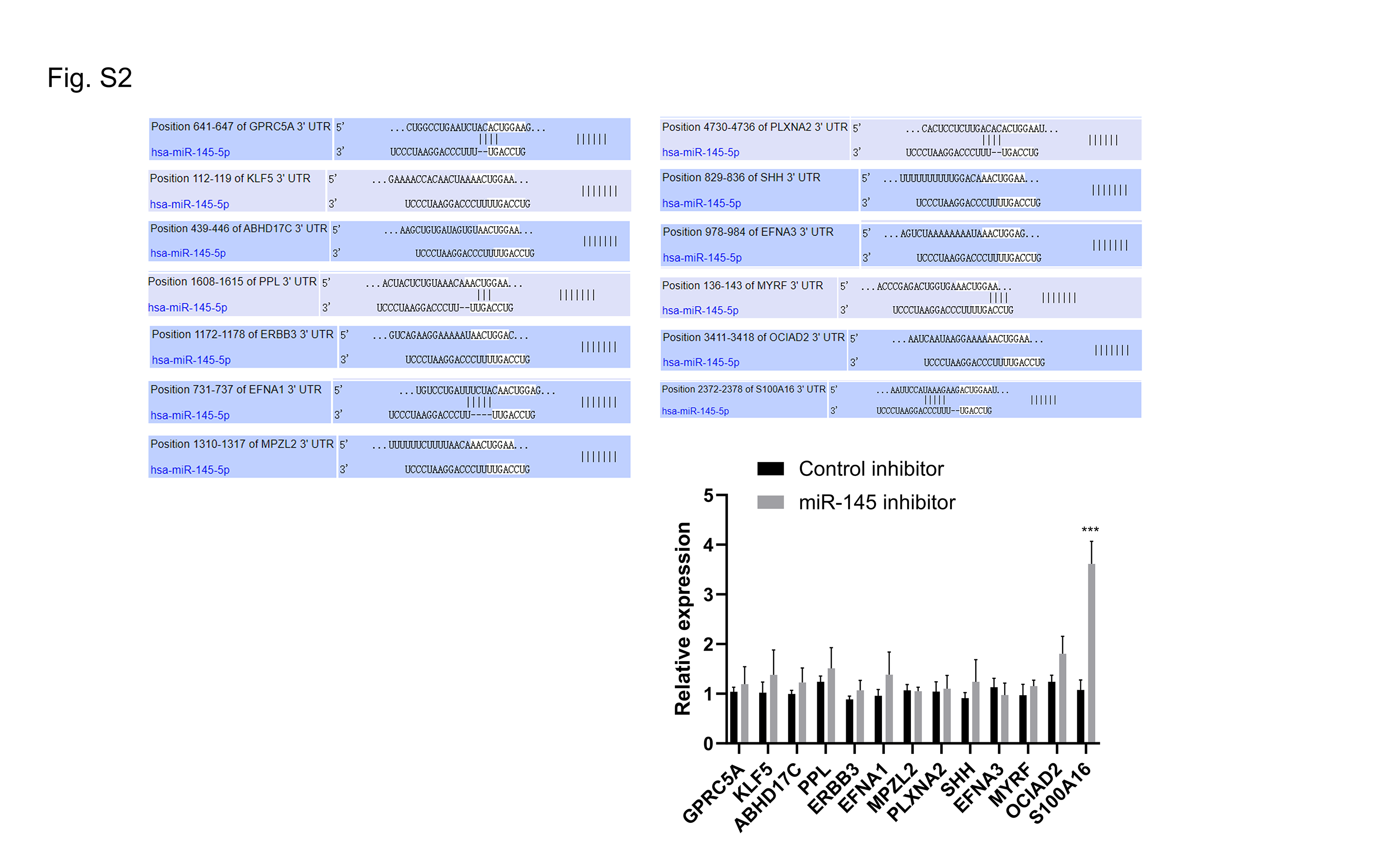

Supplement: Supplementary file 2 — Figure S2. [file JCMM-28-e18444-s001.tif]
